# Restoration of KMT2C/MLL3 in human colorectal cancer cells reinforces genome-wide H3K4me1 profiles and influences cell growth and gene expression

**DOI:** 10.1186/s13148-020-00863-z

**Published:** 2020-05-29

**Authors:** Chatarina Larsson, Lina Cordeddu, Lee Siggens, Tatjana Pandzic, Snehangshu Kundu, Liqun He, Muhammad Akhtar Ali, Nuša Pristovšek, Karin Hartman, Karl Ekwall, Tobias Sjöblom

**Affiliations:** 1 Department of Immunology, Genetics and Pathology, Uppsala University, Rudbeck Laboratory, SE-751 85 Uppsala, Sweden; 2 Department of Biosciences and Nutrition, NEO, Karolinska Institute, SE-141 83 Huddinge, Sweden; 3 Present address: School of Biological Sciences, University of the Punjab, Lahore, Pakistan; 4 Present address: The Novo Nordisk Foundation for Biosustainability, Technical University of Denmark, Kemitorvet, Building 220, 2800 Kgs. Lyngby, Denmark

**Keywords:** KMT2C, MLL3, H3K4me1, Cancer

## Abstract

**Background:**

The histone 3 lysine 4 (H3K4) monomethylase KMT2C is mutated across several cancer types; however, the effects of mutations on epigenome organization, gene expression, and cell growth are not clear. A frequently recurring mutation in colorectal cancer (CRC) with microsatellite instability is a single nucleotide deletion within the exon 38 poly-A(9) repeat (c.8390delA) which results in frameshift preceding the functional carboxy-terminal SET domain. To study effects of *KMT2C* expression in CRC cells, we restored one allele to wild type *KMT2C* in the two CRC cell lines RKO and HCT116, which both are homozygous c.8390delA mutant.

**Results:**

Gene editing resulted in increased *KMT2C* expression, increased H3K4me1 levels, altered gene expression profiles, and subtle negative effects on cell growth, where higher dependence and stronger effects of *KMT2C* expression were observed in RKO compared to HCT116 cells. Surprisingly, we found that the two RKO and HCT116 CRC cell lines have distinct baseline H3K4me1 epigenomic profiles. In RKO cells, a flatter genome-wide H3K4me1 profile was associated with more increased H3K4me1 deposition at enhancers, reduced cell growth, and more differential gene expression relative to HCT116 cells when KMT2C was restored. Profiling of H3K4me1 did not indicate a highly specific regulation of gene expression as KMT2C-induced H3K4me1 deposition was found globally and not at a specific enhancer sub-set in the engineered cells. Although we observed variation in differentially regulated gene sets between cell lines and individual clones, differentially expressed genes in both cell lines included genes linked to known cancer signaling pathways, estrogen response, hypoxia response, and aspects of immune system regulation.

**Conclusions:**

Here, KMT2C restoration reduced CRC cell growth and reinforced genome-wide H3K4me1 deposition at enhancers; however, the effects varied depending upon the H3K4me1 status of KMT2C deficient cells. Results indicate that KMT2C inactivation may promote colorectal cancer development through transcriptional dysregulation in several pathways with known cancer relevance.

## Background

Epigenetic modifiers are frequently mutated in cancer [1, 2]. Causal links to cancer development are established for some but not all of these genes. Proteins of the mixed lineage leukemia (MLL), also known as histone-lysine N-methyltransferase 2 (KMT2), family are subunits of multi-protein complexes that deposit methyl groups on lysine 4 of histone 3 through a SET-domain catalyzed histone methyltransferase activity [3]. The genes encoding the KMT2 proteins have been identified to be altered in several cancer types (reviewed in [4, 5]). The first family member to be linked to cancer development was KMT2A (MLL1), for which translocations resulting in oncogenic fusion proteins were first discovered in leukemias, and non-synonymous mutations, predominantly frameshift and nonsense, are frequently found in several solid tumor types [4, 5]. The genes encoding KMT2C (MLL3) and KMT2D (MLL4) are among the most frequently mutated in cancer [1]. These two proteins function in maintenance of histone 3 lysine 4 monomethylation (H3K4me1) levels at enhancer elements (reviewed in [6]). Analysis of clinical samples has revealed reduced *KMT2C* expression in larynx carcinoma [7], pancreatic ductal adenocarcinoma [8], and gastric cancer [9], and silencing of *KMT2C* due to promoter DNA hypermethylation has been observed in urothelial cancer [10]. The *KMT2C* gene is located on chromosome 7q36.1, which is commonly deleted in hematological malignancies [11, 12]. Deletion of *KMT2C* has also been identified in colorectal cancer (CRC) [13], and somatic mutations in *KMT2C* have been identified as potential drivers of tumorigenesis in several tumor types, including CRC [1, 14]. Missense and non-sense germline *KMT2C* variants have also been associated with cancer development in families with suspected hereditary cancer [15–18]. Of mutations present in the COSMIC database, 28.3% of *KMT2C* and 37.0% of *KMT2D* mutations, primarily frameshift and nonsense mutations, were previously found to impact the catalytic SET domain of the respective proteins [4]. A substantial proportion of mutations, notably many missense mutations, was also found in the PHD domains of KMT2C (17.1%) and KMT2D (12.9%). The mutational pattern suggests a tumor suppressor function of KMT2C which may be disrupted by differently localized mutations.

Several observations and experimental data further support the notion of *KMT2C* as a tumor suppressor gene. Forward genetic screens based on transposon mutagenesis have identified common insertion sites in the *Kmt2c* locus in mouse models of pancreatic adenocarcinoma and APC-deficient colorectal cancer development [19, 20]. Historically much focus has been on studying the function of the SET domain in cancer cells through knockout models. Specifically, biallelic knockout of the SET domain of *Kmt2c/Mll3* in mice led to formation of epithelial tumors, which supports a tumor suppressor role for the H3K4 monomethylase function of the gene [21]. More recently, PHD domain mutations in KMT2C were studied in human cells, revealing that these disrupt the interaction between KMT2C and the tumor suppressor BAP1. This induces transcriptional changes, possibly by reduced recruitment of MLL3 and UTX/KDM6A to enhancers [22], and demonstrates the value of specific study of cancer mutations to discern their functional impact in cells.

Around 15% of CRC are classified to have microsatellite instability (MSI) caused by deficiency in the mismatch repair (MMR) pathway. This subtype of tumors is prone to acquisition of frameshift mutations in short repeat sequences such as mononucleotide repeats [23, 24]. The *KMT2C* gene harbors a poly(A)9 repeat within the coding sequence of exon 38, and a recurrent frameshift mutation in this mononucleotide repeat, c.8390delA, was previously reported in 25–48% of MSI CRC cases [25, 26] and has also been shown prevalent in MSI gastric cancer [27]. The high mutation prevalence and possible loss-of-function effect [25, 26] motivated us to examine the role of *KMT2C* in CRC cells by restoring wild type KMT2C in two homozygous c. 8390delA mutant human CRC cell lines by genome editing. Comparison of cells with and without functional *KMT2C* allowed profiling of changes in H3K4me1 deposition at enhancers and changes in gene expression brought about by *KMT2C* expression, revealing that KMT2C induced a moderate global increase in H3K4me1 which affected colorectal enhancers associated with differentially expressed genes in the two isogenic cell models.

## Results

### Restoration of *KMT2C* expression in MSI CRC cells by gene targeting

**Fig. 1:**
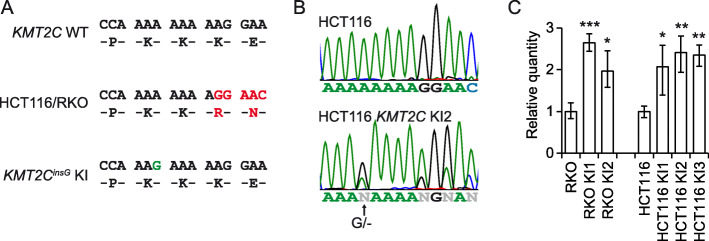
Restored *KMT2C* expression in RKO and HCT116 CRC cells with corrected c.8390delA frameshift mutation. **a** Design of isogenic cell system, showing the amino acid sequence of KMT2C at the A9 repeat of exon 38 in KMT2C wild type (WT), c.8390delA (RKO and HCT116), and knock-in (KI) cells. The frameshift mutation in the repeat is corrected by insertion of one G base (green) in the middle of the repeat to generate *KMT2C*^*insG*^ clones. This restores the protein sequence, and the G stabilizes the repeat to prevent reintroduction of mutation in the repeat through mismatch repair deficiency. The sequence frame shift is shown in red for parental HCT116 and RKO cells. **b** Sequencing of the exon 38 A9 repeat in HCT116 *KMT2C*^*insG*^ KI2 clone cDNA to verify insertion of a G base (arrow) and expression of the corrected allele. **c** Level of *KMT2C* expression measured by RT-qPCR in parental cells and *KMT2C*^*insG*^ clones. *TBP* and *HPRT1* were used as reference genes, and data were normalized to the respective parental cell line. Error bars, max and min relative quantity. Two tailed Student’s *t*-test; *P < 0.05, **P < 0.01, ***P < 0.001

A characteristic of MSI cancers is the accumulation of frameshift mutations in microsatellite repeats. The two MSI CRC cell lines RKO and HCT116 both contain homozygous c.8390delA frameshift mutations in exon 38 that are reported to lead to nonsense-mediated decay (NMD) and loss-of-function due to loss of the SET domain at the C-terminus of the protein [25, 26]. By recombinant adeno-associated virus (rAAV) mediated gene targeting in these cell lines, we aimed to restore protein function by correcting the amino acid sequence of one of the two affected alleles in each cell line to allow for full length protein expression. Since microsatellite repeats are prone to undergo frameshift mutation in MSI cancer cells, we used a strategy where we stabilized the repeat sequence by addition of a G base in replacement of the lost A base in the affected A9 repeat (Fig. 1a), as described by us previously [28]. This leads to insertion of the correct amino acid in the protein and restoration of the reading frame, and the shortened A repeat is less likely to contract frameshift mutations. Targeted clones were identified by PCR, and insertion was validated by sequencing over the target poly-A repeat in DNA (not shown) and RNA (Fig. 1b, Additional File 1, Fig. S1), which verified that the corrected allele was expressed as mRNA in all clones. Three clones with correct targeting were confirmed for the HCT116 cell line, and two clones were confirmed for the RKO cell line. We predicted restoration of the reading frame to rescue the mRNA from the NMD previously described for c.8390delA mutant *KMT2C* [25], and by RT-qPCR we detected a 2-fold increase in *KMT2C* expression in the targeted clones (Fig. 1c). This indicates that wild type *KMT2C* expression was restored in RKO and HCT116 *KMT2C*^*insG*^ cell lines and that the expression level was upregulated to the same extent in both cell lines.

### Gene expression profiling in *KMT2C*^*insG*^ clones

Monomethylation of H3K4 in combination with H3K27ac is a mark of active enhancers [29], and restoration of KMT2C function is therefore likely to increase gene transcription. We used RNA sequencing to determine the expression profile of all *KMT2C*^*insG*^ clones and the respective parental cell lines. We set a threshold for differential expression at 1.5 log2 fold up- or downregulated in at least one *KMT2C*^*insG*^ clone relative to the respective parental cell line. For validation of data, up- and downregulated genes were selected for each cell line based on fold regulation and presence of data in at least two clones per respective cell line. RT-qPCR on independently collected samples from the cell lines verified the pattern of expression detected by RNA sequencing for all of the eleven investigated genes (Additional File 1, Fig. S2). The RKO cells showed the highest number of genes (*n* = 853) with changed expression upon KMT2C restoration, with clone KI1 having 265 differentially regulated genes (DEGs) of which 57% were upregulated, and clone KI2 having 678 DEGs of which 65% were upregulated (Additional File 2, Table S1). Of the DEGs in clone 1, 90 (34%) were differentially regulated also in clone 2, which is more than expected by chance. If differential expression was random, 0–16 genes would be expected with 99% confidence to overlap between the two RKO clones (Additional File 2, Table S2). For HCT116 clones, 353 genes were affected in total, with 73, 137, and 209 DEGs in clones KI1, KI2, and KI3, respectively, and between 40–52% of these were upregulated (Additional File 2, Table S1). Also in HCT116, the number of DEGs overlapping between clones was greater than what would be expected by chance, although with lower numbers than for RKO clones (Additional File 2, Table S2). Because of H3K4me1 being an activating mark on enhancer elements, we separately analyzed the overlapping DEGs that were upregulated by *KMT2C* expression in the cell lines, revealing that also for those the overlap was greater than what would be expected from random differential expression (Additional File 2, Table S3). To investigate overlap between RKO and HCT116 cells, we included all genes that were differentially regulated in ≥ 1 KI clone. Of these, a total of 61 DEGs were common for both cell lines, but for only 29 the expression change of the affected *KMT2C*^*insG*^ clones was regulated in the same direction for both RKO and HCT116 cell lines (Additional File 3, Tables S4-5). Investigation of the expression level of these 29 genes in all individual KI clones revealed that only five genes (*ANK1*, *PRSS23*, *SAMD9*, *TSPAN1*, *WFIKKN1*) were regulated in the same direction in all the five individual *KMT2C*^*insG*^ clones. By RT-qPCR, we could validate upregulation of *ANK1*, *PRSS23*, *SAMD9*, and *TSPAN1*, however not to a significant level in all HCT116 clones, but we could not validate downregulation of *WFIKKN1* (Additional File 1, Fig. S3). Gene set overlap analysis for the respective sets of DEGs in HCT116 and RKO showed enrichment for genes in cancer-related signaling pathways such as KRAS, TNFA, and TGF-β for both RKO and HCT116 cell lines (Additional File 3, Table S6). In addition, both cell lines showed enrichment of DEGs involved in hypoxia, response to estrogen, and several immune system processes. Also for the upregulated genes estrogen response, and KRAS, TNFA, and TGF-β signaling were among the hallmark functions with significant overlap among the genes (Additional File 3, Table S7). In summary, these data showed that restoration of *KMT2C* expression in CRC cells altered the expression of hundreds of genes that, although the altered set differed between cell lines and individual *KMT2C*^*insG*^ clones, converged in common functional groups with cancer relevance.

### Profiling of H3K4 monomethylation in *KMT2C*^*insG*^ cell lines

**Fig. 2:**
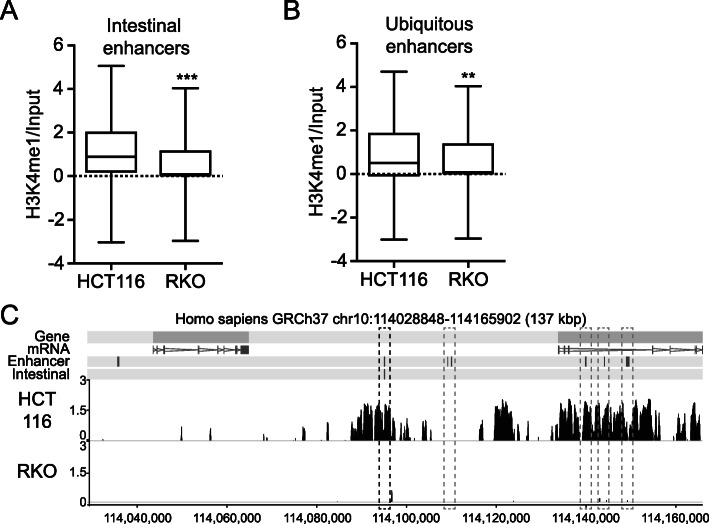
Genome-wide H3K4me1 profiling reveals differences between KMT2C deficient MSI CRC cell lines HCT116 and RKO. **a**–**b** H3K4me1 enrichment at **a** intestinal and **b** ubiquitous enhancers (**P < 0.01, ***P < 0.001). Dashed line marks 0. **c** Genome browser shot showing H3K4me1 ChIP-seq enrichment across human enhancers (gray dashed boxes), including an intestinal enhancer (black dashed box)

To profile changes in H3K4me1 upon restoration of *KMT2C* expression, we subjected cells to chromatin immunoprecipitation (ChIP) sequencing. We observed a robust genome-wide H3K4me1 profile in HCT116 despite inactivation of *KMT2C*, consistent with a previously published study demonstrating KMT2D dependent monomethylation in these cells [30]. In contrast, RKO showed a weaker and flatter H3K4me1 ChIP-seq profile with 2.2–2.4-fold fewer peaks than HCT116, depending on peak detection stringency (*p* = 1 × 10^−5^ – 1 × 10^−10^) (Additional File 1, Fig. S4a). KMT2C is predominantly reported to affect gene regulation through H3K4me1 at gene enhancers [30–32]. The weaker H3K4me1 enrichment detected in RKO cells relative to HCT116 was a global phenomenon occurring at both intestinal enhancers and ubiquitous enhancers (*n* = 447, *p* < 0.001 and *n* = 505, *p* = 0.0066, respectively) (Fig. 2a–c).

**Fig. 3:**
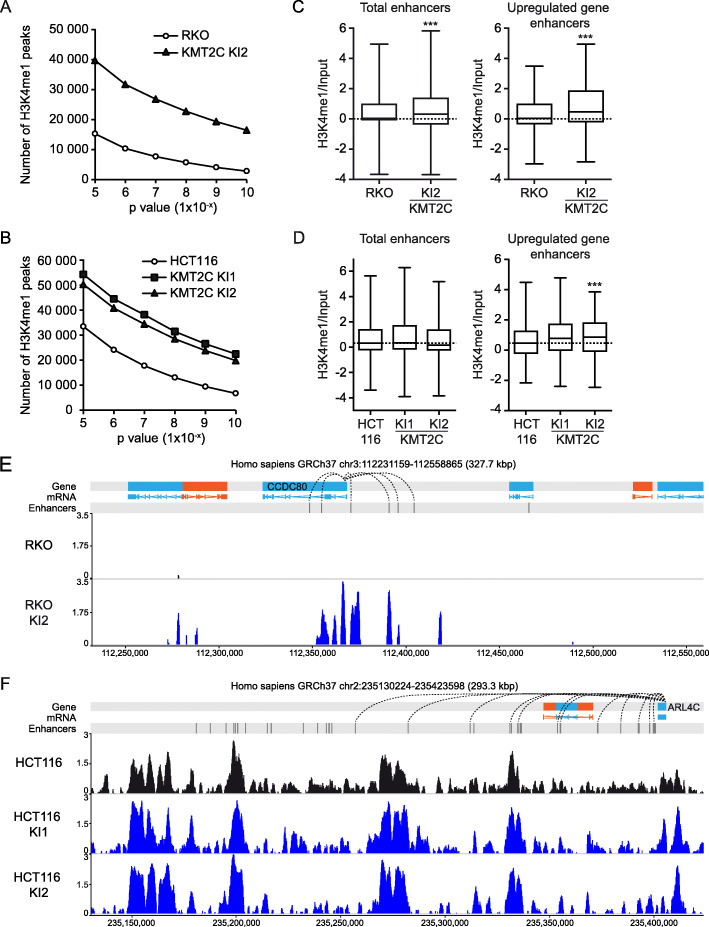
Restored *KMT2C* expression increases H3K4me1 at enhancers of upregulated genes in RKO and HCT116 cells. **a**–**b** ChIP-seq detection of H3K4me1 enriched regions at different stringencies of peak detection in RKO *KMT2C*^*insG*^ KI2 clone (**a**) and HCT116 *KMT2C*^*insG*^ KI1 and KI2 clones (**b**). **c**–**d** H3K4me1 enrichment at global human genome enhancers and at enhancers of upregulated genes in (**c**) RKO parental and *KMT2C*^*insG*^ KI2 clone and (**d**) HCT116 parental and *KMT2C*^*insG*^ clones KI1 and KI2 (***P = < 0.001). Dashed lines mark the median of the parental cells in **c**–**d**. **e** H3K4me1 signal at the *CCD80* gene, which was upregulated in RKO *KMT2C*^*insG*^ KI2 cells. **f** Detection of H3K4me1 at the chromosomal region of the *ARL4C* gene which was upregulated in HCT116 *KMT2C*^*insG*^ KI1 and KI2 clones. Enhancer-promoter associations are marked by dotted lines in **e**–**f**

Upon restoration of *KMT2C*, a 2.6–5.9 and 1.5–3.4-fold increase in H3K4me1 enriched regions was detected, respectively, for RKO and HCT116 *KMT2C*^*insG*^ cells (Fig. 3a–b). The *KMT2C* restoration-induced enrichment was stronger in RKO *KMT2C*^*insG*^ cells, which have a weaker baseline level of H3K4me1 in parental RKO cells. Global levels of H3K4me1 in *KMT2C*^*insG*^ cells and parental HCT116 and RKO cells were also estimated by immunoblotting (Additional File 1, Fig. S4b and c), independently confirming the pattern for increased H3K4me1 in *KMT2C*^*insG*^ cells, however with increases of lower magnitude than estimated by ChIP sequencing, and only at a significant level in RKO *KMT2C*^*insG*^ clone KI2. The immunoblot also validated the lower baseline H3K4me1 level in RKO cells. Using enhancers with predicted enhancer-promoter pairs, obtained from the FANTOM consortium, we measured H3K4me1 in the total FANTOM enhancer data set (*n* = 43,011) and at enhancers of genes showing upregulated expression in RKO or HCT116 *KMT2C*^*insG*^ cells, respectively (Fig. 3c–d). For RKO cells, *KMT2C* restoration resulted in increased H3K4me1 levels globally across all enhancers in the investigated *KMT2C*^*insG*^ clone (Fig. 3c; *p* < 0.001). This increase was also observed at enhancers of upregulated genes (*n* = 680, *p* < 0.001). For HCT116 cells, H3K4me1 was increased at enhancers of upregulated genes upon *KMT2C* restoration (*n* = 178) but was not apparent at enhancers globally (Fig. 3d). Examination of the H3K4me1 profiles showed that H3K4me1 was enriched at previously unmethylated enhancers in RKO cells whereas HCT116 cells had H3K4me1 already prior to *KMT2C* restoration, exemplified by enhancers of the *CCDC80* gene, which was 7-fold upregulated in RKO *KMT2C*^*insG*^ clone KI2 (Fig. 3e), and *ARL4C*, which was 4-fold upregulated in HCT116 *KMT2C*^*insG*^ clone KI3 (Fig. 3f). We examined if the additional H3K4me1 peaks detected by restoration of *KMT2C* expression in HCT116 cells reflected reinforced and more easily detected signal at sites with some H3K4me1 already present or occurred at truly new sites of H3K4me1 deposition. Consistent with the model proposed by Hu et al. [30], we found that regions that acquired H3K4me1 in *KMT2C*^*insG*^ cells showed some degree of H3K4me1 deposition in parental HCT116 cells (Additional File 1, Fig. S5). In summary, these data suggest that restoration of *KMT2C* expression affected H3K4me1 levels at enhancers. Furthermore, comparison between the two cell lines showed that genome-wide H3K4me1 was more dependent on KMT2C expression in RKO cells whereas the modest increase of H3K4me1 levels detected at the enhancers of upregulated genes in HCT116 *KMT2C*^*insG*^ clones suggests that the HCT116 cell line is less dependent on KMT2C activity to maintain H3K4me1 marks.

### Characterization of cell growth

**Fig. 4:**
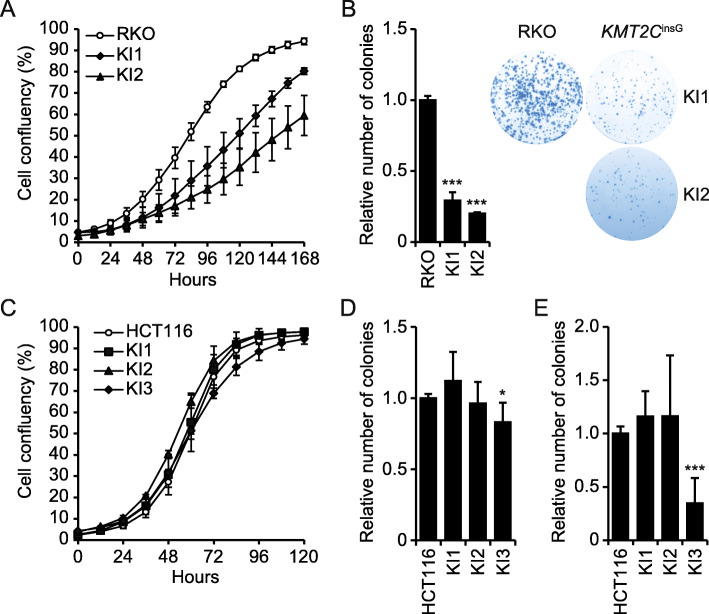
Restoration of *KMT2C* expression decreases growth of RKO cells. **a** Growth curve for RKO *KMT2C*^*insG*^ clones (KI1-2). Cell confluency was tracked by daily imaging for 1 week. Bars represent standard deviation for three replicate wells per sample (KI1, *P* < 0.05 and KI2 *P* < 0.01 for time points 48–168 h (Student’s *t*-test)). **b** Colony formation assay showing ability to form colonies from single cells for RKO *KMT2C*^*insG*^ clones. Triplicate sample wells were normalized to the average relative number of colonies for parental RKO cells. **c** Growth curve for HCT116 *KMT2C*^*insG*^ clones (KI1-3). Cell confluency was traced by daily imaging over the course of 5 days. Bars represent standard deviation for three replicate wells per sample. **d** Colony formation assay for HCT116 parental cells and *KMT2C*^*insG*^ clones. **e** Ability for anchorage independent-growth measured through the soft agar assay for HCT116 parental cells and *KMT2C*^*insG*^ clones. For **d**–**e**, all wells were normalized to the average for the respective parental HCT116 cells for each plate, and the average relative number of colonies from two independent experiments is shown for each cell line. Error bars, SD. Two tailed Student’s *t*-test; *P < 0.05, ***P < 0.001

To investigate if restoration of *KMT2C* expression affects cell growth phenotypes, we monitored cell proliferation. For RKO cells, both the restored clones showed decreased cell proliferation rate compared to parental RKO, with clones KI1 and KI2 growing ~ 10% and 30% slower than parental RKO cells, respectively (Fig. 4a). The stronger growth phenotype in clone KI2 was also accompanied by a morphological change, with cells becoming enlarged and flattened compared to the parental RKO cells (Additional File 1, Fig. S6). The altered growth phenotype of RKO *KMT2C*^*insG*^ clones was further confirmed in colony formation assays where both RKO *KMT2C*^*insG*^ clones formed markedly fewer colonies than parental RKO cells (Fig. 4b). In contrast, HCT116 *KMT2C*^*insG*^ clones did not show altered growth rate compared to parental HCT116 cells (Fig. 4c). In addition, only one of the three HCT116 *KMT2C*^*insG*^ clones (KI3) showed a trend of reduced colony forming ability in the colony formation assay (Fig. 4d). When investigating the anchorage independent growth by soft agar assays for HCT116 cells, we observed a decreased ability to grow in soft agar for the HCT116 *KMT2C*^*insG*^ KI3 clone (Fig. 4e). As we could not obtain reproducible growth of RKO cells in soft agar, we could not assess RKO cell growth by this assay. In conclusion, correction of *KMT2C* by gene editing had different effects in the two targeted cell lines. RKO cells were strongly affected by restoration of this histone methyltransferase, showing reduced growth rate, morphological changes, and compromised ability to form colonies. In contrast, with the exception of one clone that showed a tendency for decreased colony formation ability, we did not observe any alterations of the growth of HCT116 cells.

### Knockdown of *TSPAN1* and *PRSS23* in colorectal cancer cells

Next, we investigated the genes found regulated by restored KMT2C activity in RKO and HCT116 cells. Based on expression data and literature and database searches, we focused on the upregulated genes *PRSS23* and *TSPAN1.* For *PRSS23*, overexpression (average 4.9%, median 4.7% of samples) is indicated for several cancer types but no frequent mutation or copy number aberration (COSMIC database [33], v86, accessed Nov. 8 2018). The serine protease is evolutionary conserved among vertebrates and suggested involved in endothelial-mesenchymal transition regulation during cardiac development [34] and in ovarian follicle regulation [35]. The small transmembrane glycoprotein TSPAN1 was previously shown downregulated in HCT116 *KMT2D* KO cells [36]. Downregulation of *TSPAN1* has been associated with metastasis in prostate cancer [37], and knockdown inhibited the proliferation of CRC cells and their ability to migrate in an in vitro invasion assay [38]. Enrichment of H3K4me1 was observed at the enhancer of *PRSS23* in the *KMT2C*^*insG*^ KI clones for both RKO and HCT116 cells but with stronger enrichment profile in RKO cells. For *TSPAN1*, H3K4me1 enrichment was found at the gene body (Additional File 1, Fig. S7). We performed stable *PRSS23* and *TSPAN1* knockdown in the CRC cell lines DLD1 and HT-29, which do not have truncating *KMT2C* or *KMT2D* mutations (COSMIC [33], v86, 11 Oct. 2018), to study how CRC cells respond to silencing of these genes (Additional File 1, Fig. S8). Knockdown of *PRSS23* or *TSPAN1* did not affect DLD1 or HT-29 cell growth rate and growth in soft agar, or the ability for DLD1 cells to form colonies from single cells. For HT-29, cell colonies attached to poorly to the surface to be assayed. Finally, because of possible involvement with cell migration [34, 38], we performed scratch wound healing assays, where both knockdown of *PRSS23* and *TSPAN1* showed tendency to increase the wound closing capability of DLD1 cells, but not that of HT-29 cells. In summary, these data do not support a major role of *PRSS23* or *TSPAN1* upregulation in the growth phenotype detected in RKO *KMT2C*^*insG*^ cells but indicate that *PRSS23* or *TSPAN1* downregulation can affect CRC cell migration.

## Discussion

The H3K4 monomethylase KMT2C is a frequent target of mutations in CRC and other cancer types [1], and studies have demonstrated different effects of different mutations on KMT2C function [22, 39], suggesting the need for further investigation of KMT2C in tumorigenesis. Here, we restored wild type function of KMT2C in two CRC cell lines with homozygous *KMT2C* c.8390delA frameshift mutation, which is frequent in MSI cancers. No or low expression of truncated protein has been reported for this mutation previously [25, 26], and accordingly *KMT2C* expression in the parental cells was low but detectable. The general tendency for slower growth or decreased colony forming ability detected for the respective RKO and HCT116 derived *KMT2C*^insG^ KI cell lines was consistent with the suggested tumor suppressor role of KMT2C (reviewed in [5, 6]). The engineered cell lines, however, displayed changes of different magnitude, as was observed also for changes in gene expression and histone methylation patterns.

There was an overall increase in H3K4me1 following restoration of a single *KMT2C* allele, but the RKO and HCT116 cell lines varied in the amount of H3K4me1 present in the parental *KMT2C* deficient cells. Previously, from study of HCT116 cells (which express KMT2D but have *KMT2C* homozygous mutation) where *KMT2D* was knocked out, it has been proposed that KMT2C and KMT2D cooperate by targeting the same genomic regions for deposition of H3K4me1 in CRC cells [30]. In agreement with this functional redundancy model, it may be that HCT116 cells show weak effects on H3K4me1 enrichment upon *KMT2C* restoration because of having active KMT2D and a robust genome-wide profile of H3K4me1 at baseline. This fits well with the observation that in HCT116 the increased H3K4me1 enrichment in *KMT2C*^*insG*^ clones was mainly located at sites already marked by H3K4me1 in parental cells. In RKO, sequence data reported in the CCLE [40] and COSMIC [33] (v86) databases (accessed 9 Oct. 2018) indicate the presence of *KMT2D* frameshift and/or nonsense mutations. Furthermore, our RNA-seq data report lowers expression of *KMT2D* in RKO cells than in HCT116 cells (ave FPKM 1.9 for RKO vs 3.0 for HCT116). Inactivity of KMT2D may thus contribute to the lower baseline H3K4me1 level and the greater enhancement of H3K4me1 following restoration of KMT2C observed in RKO cells. Accordingly, more pronounced effects on gene expression and cell growth were also observed in RKO cells.

Although KMT2C is known to interact with certain sequence specific transcription factors, such as FOXA1 [41], based on our observations, its monomethylase activity is not highly targeted at specific genes as deposition of H3K4me1 in CRC cells where KMT2C is restored is found globally at enhancer regions, not at a specific sub-set of enhancers. Even though we detected variation in regulated gene sets between cell lines and individual KI clones, the overlap in DEGs between *KMT2C*^insG^ clones and the respective parental cell line was greater than expected if differential regulation was random, particularly in RKO cells. Furthermore, analysis of gene functions showed similarities between the differentially expressed gene sets enriched in RKO and HCT116-derived clones, identifying cell signaling pathways with well-known association to cancer as affected by *KMT2C* expression. We suggest that this could mean that *KMT2C* loss leads to general destabilization of the cells epigenomic transcriptional control rather than leading to specific downregulation of only certain target genes. This fits well with a previously proposed model for the contribution of inactivating *KMT2C* and *KMT2D* mutations to cancer development, where several mechanisms that reduce enhancer activation alter tumor suppressing gene expression [42]. Some of the variation in differential expression that was observed between clones could potentially stem from the clonal selection procedures used to isolate the targeted clones [43], whereas there is low risk for simultaneous off-target integrations of the rAAV vector with correct rAAV-mediated gene targeting [44]. Interestingly, given that KMT2C has been found to regulate estrogen-dependent HOX family gene transcription in chorocarcinoma placenta cells [45–47], and identified an estrogen receptor α (ERα) coactivator in breast cancer cells [48], we observed differential regulation of estrogen response genes. Among these were PRSS23, which was upregulated in all *KMT2C*^*insG*^ clones and for which H3K4me1 was enriched at enhancers. Levels of PRSS23 correlate with ERα levels in breast cancer and are upregulated by ERα promoter binding [49]. Our data thus suggest that KMT2C may be important for regulation of estrogen signaling in CRC tumorigenesis, but unlike previous reports in breast cancer [49], we saw minor effects of *PRSS23* knockdown on CRC cell proliferation, suggesting the possibility that other KMT2C regulated genes are more important for promotion of CRC tumorigenesis following loss of KMT2C.

Finally, in this paper we have studied a frameshift mutation in exon 38, which precedes the SET domain, FYRC and FYRN domains as well as one PHD-like domain. We focused on investigating effects on enhancer H3K4me1 changes and differential gene expression. From studies of catalytic dead vs knocked out *KMT2C* in mouse embryonic stem cells, it has been suggested that not all functions of KMT2C and KMT2D are dependent on the catalytic function of the SET domain [50]. Thus, we cannot exclude the possibility that *KMT2C* c.8390delA mutations contribute to cancer development through other, yet to be defined, mechanisms.

## Conclusions

This is the first study where an endogenous *KMT2C* frameshift mutation is functionally restored in CRC cells. Heterozygous *KMT2C* knock in led to a general tendency of reduced colony formation ability. Gene expression profiling in *KMT2C*^insG^ cells did not point to altered expression of specific gene sets but rather to a general transcriptional dysregulation that affected processes or pathways that are known players in cancer development, lending support to this as a plausible mechanism for selection of *KMT2C* mutations during cancer development. The striking difference in H3K4me1 genome-wide profile between HCT116 and RKO cell lines demonstrates how individual mutations can yield different molecular phenotypes. Molecularly, this is not particularly surprising due to the redundancy of molecular pathways in mammalian cells whereby most genes are regulated by several transcription factors, coactivators, and repressors. Thus, when one transcriptional coactivator such as KMT2C is lost, it does not lead to a complete failure or suppression of gene expression or even of H3K4me1 deposition. Future studies should address if in clinical settings KMT2C-deficient colorectal cancers have distinct H3K4me1 phenotypes. This may pave the way for personalized treatments whereby patients displaying KMT2C loss and aberrant H3K4me1 epigenomic profiles may respond well to treatments that restore H3K4me1 while KMT2C wild type CRC patients with normal H3K4me1 profiles would likely be unaffected.

## Materials and methods

### *KMT2C* targeting construct

Primers used for PCR are listed in Additional File 2, Table S8. The *KMT2CinsG*-rAAV gene targeting vector was designed to contain a guanine instead of an adenosine in the fourth position of the A(9) repeat in exon 38. The 5’ and 3’ homology arm (HA) sequences were amplified from HCT116 gDNA with Phusion High-Fidelity DNA polymerase (Finnzyme, Espoo, Finland) with *attB* tagged primers 1–4 and gel purified. The HA sequences and the selection cassette were cloned into the pAAV-Dest destination vector using the Gateway system (Invitrogen, Carlsbad, CA, USA) as described [51]. Briefly, 100 ng each of the amplified 5’ (HA1) and 3’ (HA2) HAs were recombined with 150 ng of pDONR^TM^P1-P2 or pDONR^TM^P3-P4, respectively, using BP Clonase II, generating pEntry-HA1 and pEntry-HA2. Positive clones were identified by PCR with primers 5–6. The G nucleotide was inserted within the A(9) repeat of the 3’ HA sequence inserted in pDONR^TM^P3-P4 using the QuickChange II mutagenesis kit (Stratagene, San Diego, CA, USA) with primers 7–8, and the insertion was confirmed by Sanger sequencing. Next, 10 fmol each of pEntry-HA1, the selection-cassette encoding entry vector pBUOY.SA.IRES.Neo.pA, and pEntry-HA2 were recombined with 15 fmol pAAV-Dest vector using LR Clonase II. The presence and orientation of fragments was confirmed by PCR and Sanger sequencing using LR screening primers 9–12. The *KMT2CinsG*-rAAV virus particles were produced by transfection of 70% confluent AAV-293 cells (Stratagene, cultured in DMEM, 10% FBS and 1% penicillin/streptomycin (PEST) (all from Gibco/Life Technologies, Carlsbad, CA, USA)) with Lipofectamine (Invitrogen) and 5 μg each of pAAV-RC, pHELPER (Stratagene), and the targeting construct, with harvesting of cell lysate after 48 h as described [52].

### Cell lines and *KMT2C* targeting

The human colorectal cancer cell lines RKO (CRL-2577, ATCC, Manassas, VA, USA) and HCT116 (CCL-247, ATCC) were cultured in McCoy’s 5A (modified) medium (Gibco) with 10% FBS and 1% PEST. Cells were transfected with *KMT2CinsG*-rAAV as described [52] and selected for 2 weeks at limiting dilution in the presence of 0.8 mg/ml (RKO) or 0.4 mg/ml (HCT116) Geneticin (Gibco). Single-cell clones with site-specific integration of the targeting vector were identified by PCR (primers 13–18), and the insertion of a G in the A9 repeat, as well as the integrity of the sequence surrounding the insertion site, was confirmed by Sanger sequencing. The IRES *neo* selection cassette was removed by Ad-Cre virus (Vector Biolabs, Malvern, PA, USA) infection as described [52], and single-cell clones identified by PCR (primers 19–20) to lack selection cassette were verified by their inability to grow in the presence of Geneticin. The *KMT2CinsG* clones were validated by sequencing of the exon 38 A(9) repeat, and the inserted G was verified to be expressed by Sanger sequencing of RT-PCR products. Parental and isogenic cell lines were authenticated by STR profiling at ATCC (2017) and verified free from mycoplasma using the MycoAlert mycoplasma detection kit (Lonza, Basel, Switzerland) prior to RNA and ChIP sequencing.

### Generation of stable *PRSS23* and *TSPAN1* knockdown cell lines by lentiviral transduction of shRNAs

The cell lines DLD1 and HT-29 (HTB-38, ATCC) were cultured as described for HCT116 and RKO above. Lentiviral transductions were performed with SMARTvector lentiviral particles (Horizon/Dharmacon) (Additional File 2, Table S9). The day before transduction, 50,000 cells were plated in each well of a 24-well plate. The next day, the plating medium was removed, and 250 μl of virus diluted in normal growth medium with 7.5 mg/ml Sequa-Brene (Sigma Aldrich) was added to each well. After 24 h incubation at 37 °C, virus-containing media were replaced with fresh complete growth media. After 48 h incubation, the pool of cells containing the shRNA of interest was selected by addition of 1 μg/ml puromycin (Gibco) to the growth medium. After expansion for 2–3 passages under selection with puromycin, knockdown efficiencies were determined by qPCR.

### Cell morphology, growth, and migration

Growth curves were generated by culturing of cells in plates imaged in real-time inside an IncuCyte HD instrument, recording cell confluency every 12 h. For colony formation assays, 500 cells (HCT116, DLD1) or 1000 cells (RKO) plated in triplicate wells of 6-well plates were stained with 5% methylene blue in methanol 10–14 days after seeding. Soft agar assays were performed in 6-well plates with a 1-ml top layer of 0.3% (RKO, DLD1, HT-29) or 0.35–0.4% (HCT116) low gelling temperature agarose (A9414, Sigma Aldrich, St. Louis, MO, USA) with 1000–1250 resuspended cells, which was overlaid onto 1 ml of a bottom layer of solid 1% (RKO) or 0.8% (HCT116, DLD1, HT-29) low gelling temperature agarose. After solidification, the top layer was covered with 1 ml of fresh culture medium. Colonies were stained with 0.05% crystal violet after 2–3 weeks. For study of cell migration, confluent cell monolayers in 6 well plates were scratched with a 200-μl pipet tip, washed with fresh medium at least three times, and imaged at 0 h and after 72 h culturing. The TScratch software [53] was used to calculate the open wound area at defined spatial areas of the scratch.

### RNA sequencing

The integrity and concentration of total RNA, isolated using the RNeasy mini kit with on-column DNAse digestion (Qiagen, Hilden, Germany), was determined on a Bioanalyzer 2100 instrument (Agilent, Santa Clara, CA, USA) using the RNA 6000 nano chip. Samples were sequenced on the Ion Proton system (Ion Torrent/Life Technologies) at the Uppsala Genome Center at SciLife Lab NGI Uppsala. The sequencing reads were aligned to the human genome hg19 assembly [54] using the Tophat2 software (version 2.0.4) [55]. Quantification of gene expression levels and identification of differential expression were performed using the Cufflinks software (version 2.1.1) [56]. Among the most deregulated genes, 6 were chosen for RT-qPCR validation for each cell line, respectively, although one primer pair failed for the RKO cell line. Overlap with the Hallmarks gene set [57] using the Molecular Signatures Database (MSigDB) [58, 59] was used for functional classification and enrichment analysis of regulated genes.

### RT-qPCR

Primers for qPCR are listed in Additional File 2, Table S10. All primers were evaluated by construction of five step 1:5 dilution standard curves. By stability evaluation using the Cotton EST database RefFinder tool [60] across all independent isogenic clones and the respective parental RKO and HCT116 cell lines, TBP and HPRT1 were selected as endogenous reference genes. The cDNA was synthesized from total RNA using the Maxima H minus First Strand cDNA synthesis kit (Thermo Scientific) using the supplied random primers. Technical triplicate 20 μl qPCR reaction with 1× Maxima SYBR Green/ROX qPCR Master Mix (Thermo Scientific) and 0.3 μM of each primer were run on the StepOne Real-Time PCR system (Applied Biosystems, Foster City, CA, USA). Each assay included no-template controls, and each RNA sample was assessed for gDNA contamination by cDNA synthesis reactions where reverse transcriptase was omitted. For assessment of lentiviral knockdown efficiencies for *TSPAN1* and *PRSS23*, all cDNAs were prepared directly from cell lysates using the Cell-to-Ct kit (Life Technologies), and specific TaqMan assays (ThermoFisher Scientific; Additional File 2, Table S7) were used to measure gene expression levels.

### ChIP sequencing

HCT116 cells and RKO cells were fixed in 1% paraformaldehyde for 8 min, and ChIP was performed using the iDeal ChIP-seq kit (Diagenode, Liege, Belgium) according to the manufacturer’s instructions. Chromatin extracts from one million cells were sonicated to yield 100 to 500 bp fragments using a Bioruptor Pico sonication device (Diagenode) on high power for two rounds of 6 cycles with 30 s on 30 s off at 4 °C. Fragmentation was verified with agarose gel electrophoresis. ChIP-seq was performed using 1 μg of H3K4me1 antibody (ab8895, Abcam) per ChIP. After reverse cross-linking overnight at 65 °C, DNA was extracted using the Qiagen MinElute PCR purification kit in 15 μl of elution buffer. Libraries were prepared for sequencing using the NEBNext® ChIP-Seq Library Prep Reagent Set for Illumina® and sequenced on a HiSeq 2500 System (Illumina).

### Analysis of ChIP-seq

ChIP-seq data in HCT116 and RKO samples were aligned using Bowtie 2. BAM files were uploaded into Seqmonk for further analysis [61]. Peak calling was performed with MACS peak builder at different *p* values as indicated. Ubiquitous and colorectal specific enhancers were obtained from the FANTOM 5 project [62]. FANTOM5 enhancers were identified by bidirectional transcription using the capped analysis of gene expression technique [63]. H3K4me1 signal was quantified by calculating the log2 of the reads per million for each sample relative to input (ChIP/Input). Genome browser shots were produced using Seqmonk by detecting the enrichment over input across selected regions with smoothing for adjacent 5 probes. To examine the enhancers of differentially expressed genes, we identified enhancers from the FANTOM5 project that are predicted to regulate each of the differentially expressed genes. Differences between means were examined by the Students *t*-test or ANOVA as indicated.

### Western blot

Histone extracts were prepared from 1 × 10^7^ cells using the Histone Extraction kit (Abcam, ab113476). Equal amounts of proteins were separated on NuPAGE 4–12% Bis-Tris protein gels, transferred onto nitrocellulose membranes using the iBlot system (Invitrogen), and probed with anti-H3K4me1 (ab8895, Abcam, 1:500) and anti-H3 (ab1719, Abcam, 1:3000). Immunoreactive proteins were visualized using SuperSignal West Femto Chemiluminescent Substrate (Thermo Scientific) and Amersham Imager 680 (GE Healthcare). The ratio of H3K4me1 to total H3 was quantified by densitometric analysis using ImageJ [64].

## Supplementary information

**Additional file 1: Figure S1.** Validation of *KMT2C* gene targeting in knock-in clones. **Figure S2.** RNA sequencing data for RKO and HCT116 *KMT2C*^insG^ clones were validated by RT-qPCR. **Figure S3.** The genes ANK1, PRSS23, SAMD9 and TSPAN1 were upregulated in *KMT2C*^*insG*^ HCT116 and RKO cells. **Figure S4.** Detection of H3K4me1 in HCT116 and RKO cells. **Figure S5.** Detection of H3K4me1 enriched regions is enhanced at sites of existing H3K4me1 in HCT116 *KMT2C*^*insG*^ KI clones. **Figure S6.** Restoration of *KMT2C* expression affects the morphology of RKO cells. **Figure S7.** Level of H3K4me1 at genomic regions of *PRSS23* and *TSPAN1* genes. **Figure S8.** Knockdown of *TSPAN1* and *PRSS23* in colorectal cancer cells has minor effects on cell growth.

**Additional file 2: Table S1.** Summary of genes differentially regulated in RKO and HCT116 following restoration of *KMT2C* expression. **Table S2.** Differentially regulated genes in RKO and HCT116 *KMT2C*^*insG*^ clones observed to overlap or expected to overlap by chance if regulation is random. **Table S3.** Upregulated genes in RKO and HCT116 *KMT2C*^*insG*^ clones observed to overlap or expected to overlap by chance if regulation is random. **Table S8.** PCR primer sequences. **Table S9.** The shRNA lentiviruses and TaqMan probes used for stable knockdown cell line generation. **Table S10.** Primers for RT-qPCR with SYBR Green detection.

**Additional file 3: Table S4.** Genes differentially expressed more than 1.5 log2 fold in RKO cells following restoration of *KMT2C* expression. **Table S5.** Genes differentially expressed more than 1.5 log2 fold in HCT116 cells following restoration of *KMT2C* expression. **Table S6.** Overlap analysis with the MSigDB Hallmarks gene set for genes differentially regulated >1.5 log2 fold by restoration of *KMT2C* expression in RKO and HCT116 cells. **Table S7.** Overlap analysis with the MSigDB Hallmarks gene set for genes upregulated >1.5 log2 fold by restoration of *KMT2C* expression in RKO and HCT116 cells.

**Additional file 4.** Uncropped gels for Figure S1

## Data Availability

The RNA sequencing and ChIP-seq datasets generated and analyzed during this study are available in the NCBI GEO data repository [65] with accession numbers GSE131507 [66] and GSE131755 [67], respectively. All additional data generated and/or analyzed during this study are included in this published article and its supplementary information files.

## Abbreviations

ChIP: Chromatin immunoprecipitation
DEG: Differentially regulated genes
H3K4me1: Histone 3 lysine 4 monomethylation
KMT2: Histone-lysine N-methyltransferase 2
MLL: Mixed lineage leukemia
MSI: Microsatellite instability
NMD: Nonsense-mediated mRNA decay
rAAV: Recombinant adeno-associated virus
