# Supplementary material for: Restoration of KMT2C/MLL3 in human colorectal cancer cells reinforces genome-wide H3K4me1 profiles and influences cell growth and gene expression
Source: Clin Epigenetics. 2020 May 29;12:74. doi: 10.1186/s13148-020-00863-z (PMC7257146; doi:10.1186/s13148-020-00863-z)
Supplement: Supplementary file 1 — Additional file 1: Figure S1. Validation of KMT2C gene targeting in knock-in clones. Figure S2. RNA sequencing data for RKO and HCT116 KMT2CinsG clones were validated by RT-qPCR. Figure S3. The genes ANK1, PRSS23, SAMD9 and TSPAN1 were upregulated in KMT2CinsG HCT116 and RKO cells. Figure S4. Detection of H3K4me1 in HCT116 and RKO cells. Figure S5. Detection of H3K4me1 enriched regions is enhanced at sites of existing H3K4me1 in HCT116 KMT2CinsG KI clones. Figure S6. Restoration of KMT2C expression affects the morphology of RKO cells. Figure S7. Level of H3K4me1 at genomic regions of PRSS23 and TSPAN1 genes. Figure S8. Knockdown of TSPAN1 and PRSS23 in colorectal cancer cells has minor effects on cell growth. [file 13148_2020_863_MOESM1_ESM.pdf]

ADDITIONAL FILE 1

SUPPLEMENTARY FIGURES

Restoration of KMT2C/MLL3 in human colorectal cancer cells reinforces genome wide H3K4me1 profiles and influences cell growth and gene expression

Chatarina Larsson, Lina Cordeddu, Lee Siggens, Tatjana Pandzic, Snehangshu Kundu, Liqun He, Muhammad Akhtar Ali, Nuša Pristovšek, Karin Hartman, Karl Ekwall, Tobias Sjöblom

Figure S1. Validation of *KMT2C* gene targeting in knock-in clones.

Figure S2. RNA sequencing data for RKO and HCT116 *KMT2C*^{insG} clones were validated by RT-qPCR.

Figure S3. The genes ANK1, PRSS23, SAMD9 and TSPAN1 were upregulated in *KMT2C*^{insG} HCT116 and RKO cells.

Figure S4. Detection of H3K4me1 in HCT116 and RKO cells.

Figure S5. Detection of H3K4me1 enriched regions is enhanced at sites of existing H3K4me1 in HCT116 *KMT2C*^{insG} KI clones.

Figure S6. Restoration of *KMT2C* expression affects the morphology of RKO cells.

Figure S7. Level of H3K4me1 at genomic regions of *PRSS23* and *TSPAN1* genes.

Figure S8. Knockdown of *TSPAN1* and *PRSS23* in colorectal cancer cells has minor effects on cell growth.

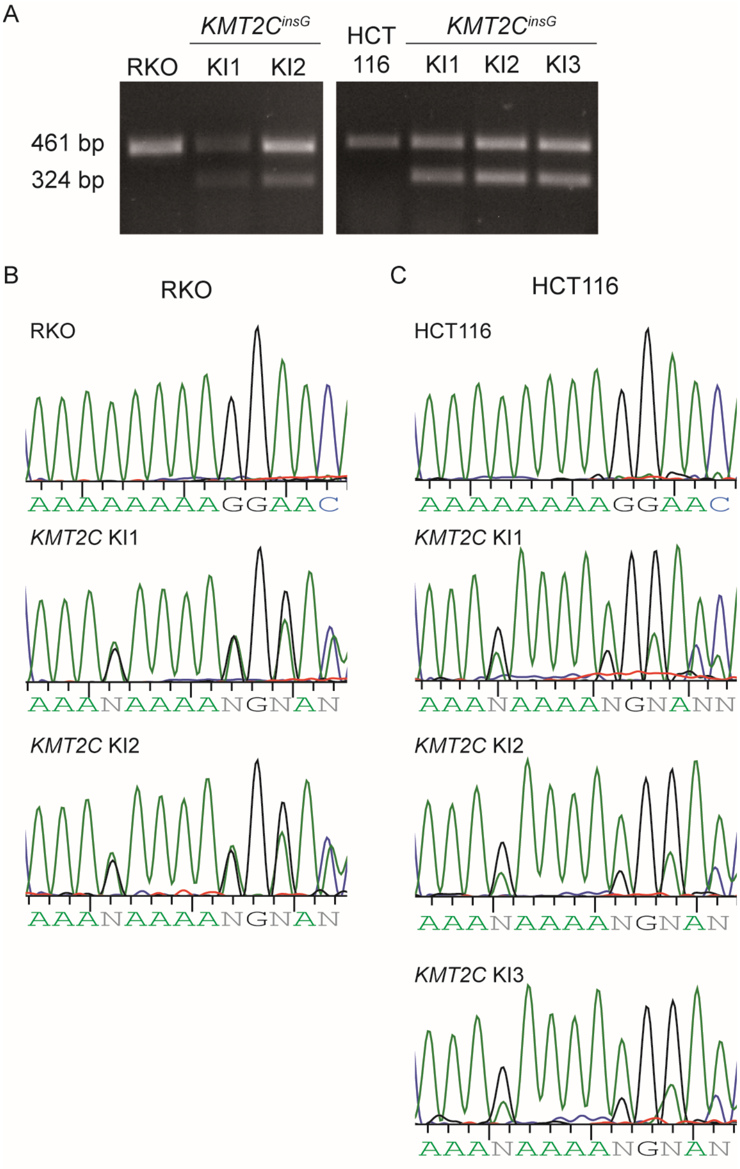

Figure S1. Validation of *KMT2C* gene targeting in knock-in clones. *A*. PCR confirmation of **t**he presence of non-targeted *KMT2C* allele (461 bp) and *KMT2C* allele edited by insertion of a G base (324 bp) in the genome of RKO and HCT116 cells (insG). The difference in PCR product length between non-targeted and targeted alleles is the result of the exchange of the 233 bp intronic sequence separating the two homology arm target sequences with 96 bp that remain of the targeting construct following excision of the selection cassette using adeno cre virus after insertion of the construct in the genome. Negative controls with H_2O were included in the PCR assay. *B-C*. Sanger sequencing of the exon 38 A9 repeat in cDNA from RKO (*B*) and HCT116 (*C*) *KMT2C*^{insG} clones validates expression of the allele with the inserted G nucleotide.

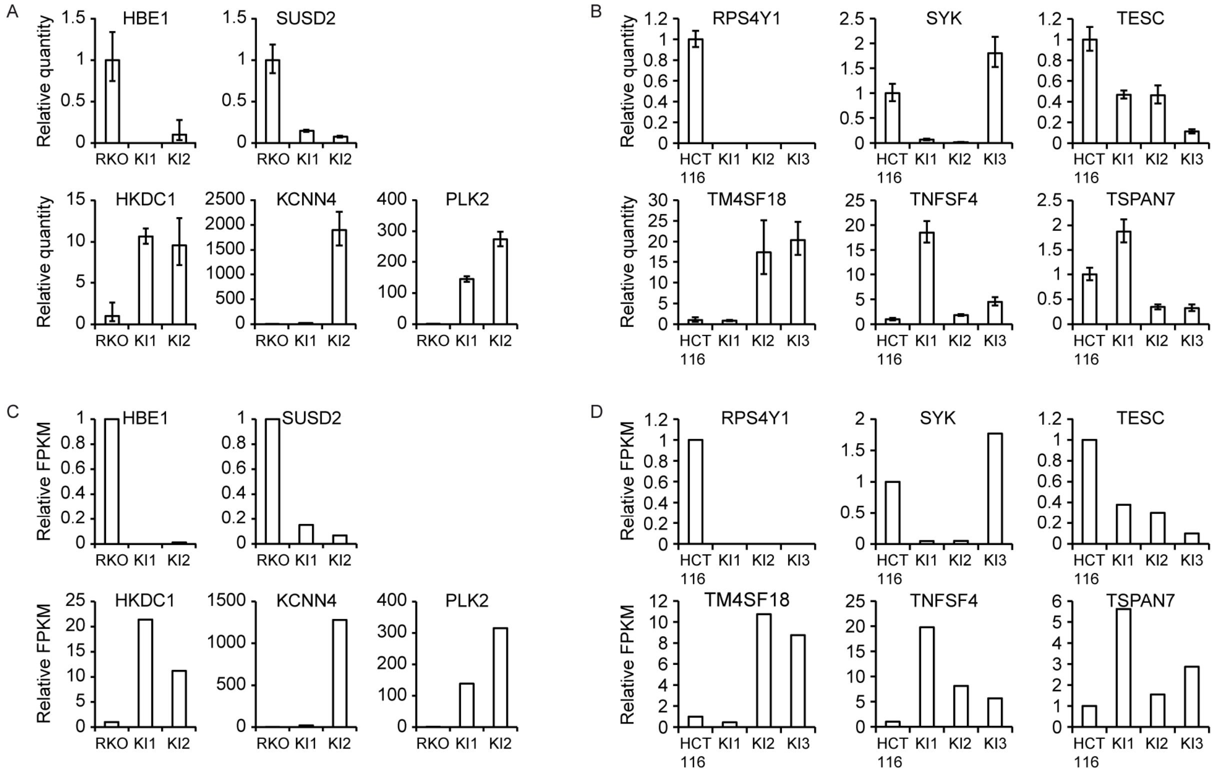

Figure S2. RNA sequencing data for RKO and HCT116 *KMT2C*^{insG} clones were validated by RT-qPCR. *A-B*. Relative expression levels of genes found differentially regulated upon restoration of *KMT2C* expression were quantified by RT-qPCR for independent samples from RKO (A) and HCT116 (B) *KMT2C*^{insG} cell lines. Gene expression was normalised to *HPRT1* (A) and *TBP* (B) expression in the respective parental cell lines. Error bars represent maximum and minimum relative expression for each cell line. Parental RKO and HCT116 cells were run with biological triplicate samples, *KMT2C*^{insG} clones were single samples. Technical triplicates were run for all samples. (*C-D*) Relative FPKM values from RNA sequencing are shown for comparison for RKO (C) and HCT116 (D) parental and *KMT2C*^{insG} cell lines.

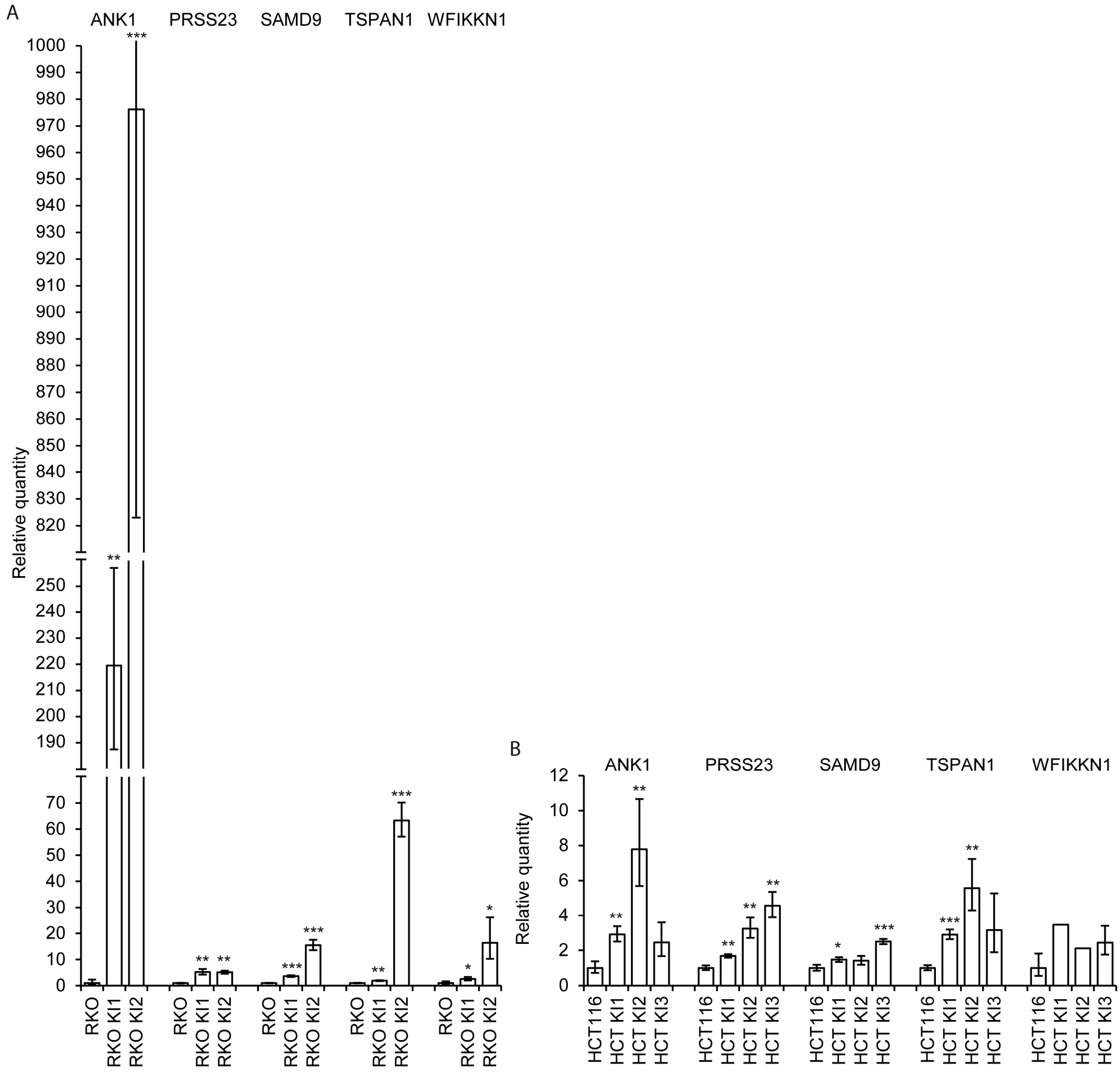

Figure S3. The genes ANK1, PRSS23, SAMD9 and TSPAN1 were upregulated in *KMT2C[insG]* HCT116 and RKO cells. Relative expression levels of genes found differentially regulated by RNA-seq upon restoration of *KMT2C* expression were quantified by RT-qPCR for independent samples from RKO (A) and HCT116 (B) *KMT2C[insG]* cell lines. Gene expression was normalised to *HPRT1* (A) and *TBP* (B) expression in the respective parental cell lines. Error bars represent maximum and minimum relative expression for each cell line. Technical triplicates were run for all samples. Two tailed student's t-test; *P<0.05, **P<0.01, ***P<0.01. Note split y-axis in panel A, due to very low expression of *ANK1* in RKO cells.

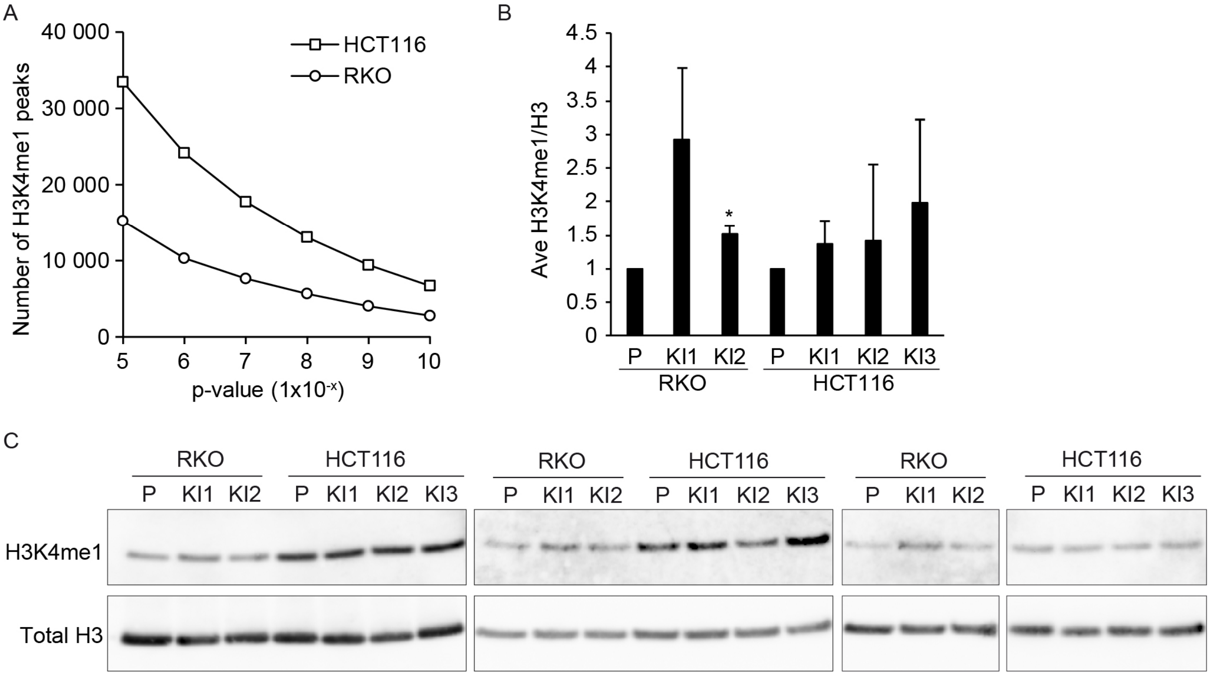

Figure S4. Detection of H3K4me1 in HCT116 and RKO cells. (A) H3K4me1 enriched regions were detected by ChIP sequencing in parental HCT116 and RKO cell lines using MACS with different p-value thresholds. (B) Quantification of H3K4me1 by immunoblotting. The ratio of H3K4me1 to total H3 in each *KMT2C*^{insG} clone (KI) is shown normalized to the respective parental cell line (P). Bars – standard deviation, n=3. Two tailed student's t-test; *P=0.015. (C) Images of the immunoblots for H3K4me1 and H3 used for quantification.

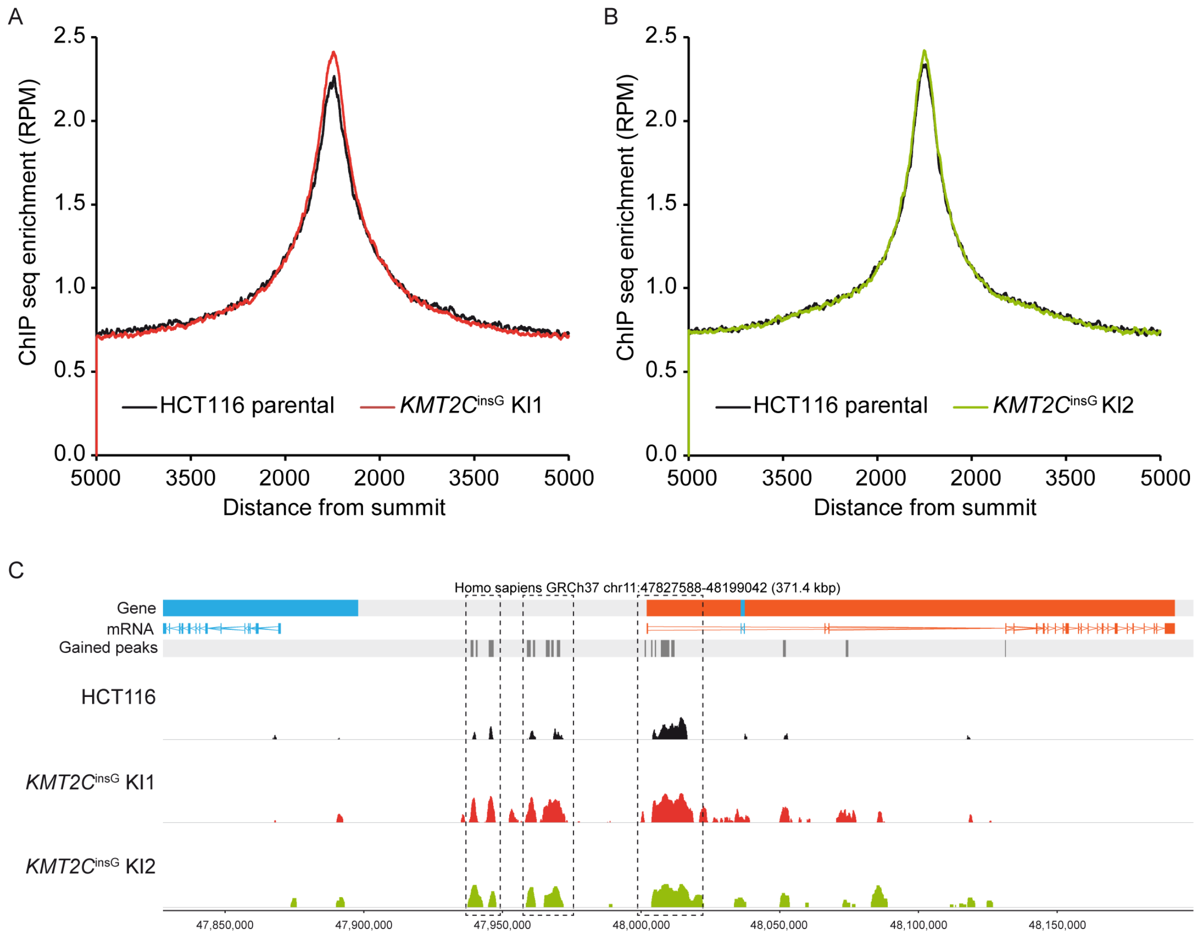

Figure S5. Detection of H3K4me1 enriched regions is enhanced at sites of existing H3K4me1 in HCT116 *KMT2C^{insG}* KI clones. *A-B*. Regions that gained H3K4me1 in HCT116 *KMT2C^{insG}* KI clones display strong H3K4me1 enrichment in parental HCT116 despite loss of KMT2C activity in these cells. *C*. Examples of regions that gain incremental H3K4me1 upon ChIP-seq detection showing existing H3K4me1 signal in the parental HCT116 cells prior to *KMT2C^{insG}* knock in.

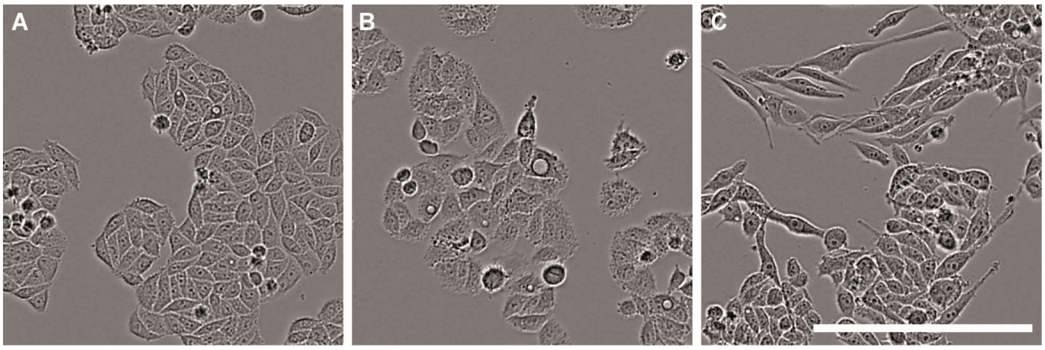

Figure S6. Restoration of *KMT2C* expression affects the morphology of RKO cells. Representative images of live cells during culture under normal growth conditions (10% FBS, 5% CO_2) in an IncuCyte HD instrument. *A*. Parental RKO cells. *B*. RKO *KMT2C*^{insG} KI1. *C*. RKO *KMT2C*^{insG} KI2. Scale bar 200 µm.

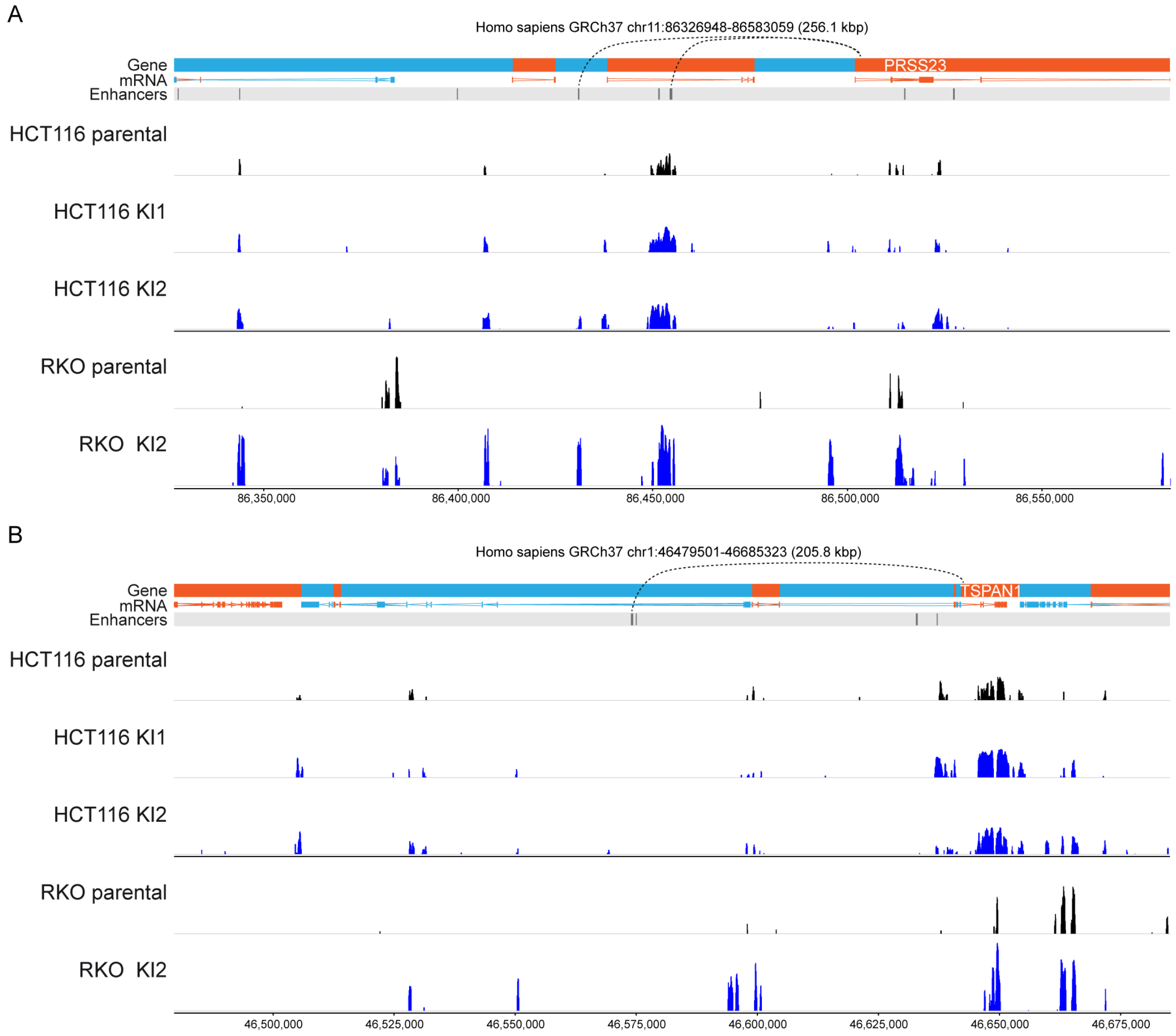

Figure S7. Level of H3K4me1 at genomic regions of *PRSS23* and *TSPAN1* genes. *A*. Peaks from H3K4me1 ChIP-seq detected on chromosome 11 near the *PRSS23* gene which is upregulated by knock-in of *KMT2C* in HCT116 and RKO cells. *B*. Peaks from H3K4me1 ChIP-seq detected on chromosome 1 near the *TSPAN1* gene which is upregulated by knock-in of *KMT2C* in HCT116 and RKO cells. Permissive enhancers that are associated with regulation of the respective genes are marked by dotted lines linking them to the genes.

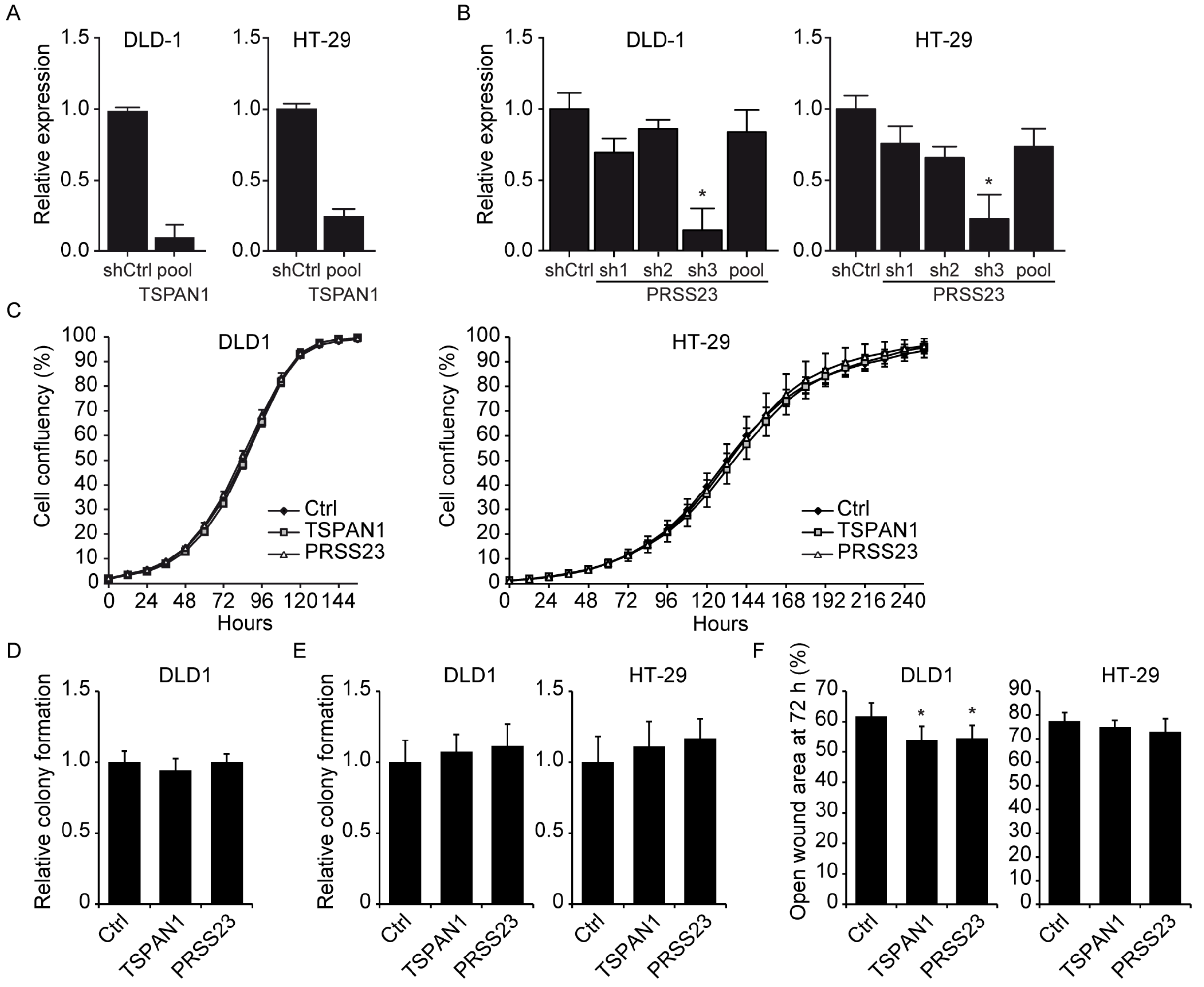

Figure S8. Knockdown of *TSPAN1* and *PRSS23* in colorectal cancer cells has minor effects on cell growth. *A-B.* Relative expression of *TSPAN1* (*A*) and *PRSS23* (*B*) in DLD1 and HT-29 CRC cell lines measured by Taqman RT-qPCR after shRNA knockdown using one or a pool of 3 specific shRNAs per gene. Error bars – SD. *C.* Growth curve for DLD1 (left) and HT-29 (right) cells expressing shRNA control or shRNA for *TSPAN1* or *PRSS23* knockdown showing cell confluency tracked through daily imaging. Bars - SD for three replicate wells. *D-E.* Colony formation assay (*D*) and soft agar colony formation assay (*E*) for DLD1 cells expressing shRNA control or shRNA for *TSPAN1* or *PRSS23* knockdown. Triplicate sample wells were normalized to the average relative number of colonies for DLD1 shRNA control cells. *F*. Wound closing measured by scratch assay for DLD1 and HT-29 cells expressing shRNA control or shRNA for *TSPAN1* or *PRSS23* knockdown. Percent wound area remaining open was calculated from the open wound area measured at 0 h and 72 h after scratching. Error bars - SD. Two tailed Student's t-test; * P<0.05. All experiments were repeated at least twice. shRNA pool was used for *TSPAN1* knockdown and shRNA3 was used for *PRSS23* knockdown in C-F.
