# Supplementary material for: Restoration of KMT2C/MLL3 in human colorectal cancer cells reinforces genome-wide H3K4me1 profiles and influences cell growth and gene expression
Source: Clin Epigenetics. 2020 May 29;12:74. doi: 10.1186/s13148-020-00863-z (PMC7257146; doi:10.1186/s13148-020-00863-z)
Supplement: Supplementary file 2 — Additional file 2: Table S1. Summary of genes differentially regulated in RKO and HCT116 following restoration of KMT2C expression. Table S2. Differentially regulated genes in RKO and HCT116 KMT2CinsG clones observed to overlap or expected to overlap by chance if regulation is random. Table S3. Upregulated genes in RKO and HCT116 KMT2CinsG clones observed to overlap or expected to overlap by chance if regulation is random. Table S8. PCR primer sequences. Table S9. The shRNA lentiviruses and TaqMan probes used for stable knockdown cell line generation. Table S10. Primers for RT-qPCR with SYBR Green detection. [file 13148_2020_863_MOESM2_ESM.pdf]

ADDITIONAL FILE 2

SUPPLEMENTARY TABLES 1, 2, 3, 8, 9, 10

Restoration of KMT2C/MLL3 in human colorectal cancer cells reinforces genome wide H3K4me1 profiles and influences cell growth and gene expression

Chatarina Larsson, Lina Cordeddu, Lee Siggens, Tatjana Pandzic, Snehangshu Kundu, Liqun He, Muhammad Akhtar Ali, Nuša Pristovšek, Karin Hartman, Karl Ekwall, Tobias Sjöblom

Table S1. Summary of genes differentially regulated in RKO and HCT116 following restoration of *KMT2C* expression.

Table S2. Differentially regulated genes in RKO and HCT116 *KMT2C*^{insG} clones observed to overlap or expected to overlap by chance if regulation is random.

Table S3. Upregulated genes in RKO and HCT116 *KMT2C*^{insG} clones observed to overlap or expected to overlap by chance if regulation is random.

Table S8. PCR primer sequences.

Table S9. The shRNA lentiviruses and TaqMan probes used for stable knockdown cell line generation.

Table S10. Primers for RT-qPCR with SYBR Green detection.

Table S1. Summary of genes differentially regulated in RKO and HCT116 following restoration of *KMT2C* expression.

**Cell line**	**Clone**	**Number of regulated genes** (>1.5 log2 fold)	**Upregulated** (%)	**Total number of regulated genes per cell line**
HCT116	KI1	73	40	353
	KI2	137	52	
	KI3	209	50	
RKO	KI1	265	57	853
	KI2	678	65	

Table S2. Differentially regulated genes in RKO and HCT116 $\mathit{KMT2C}^{insG}$ clones observed to overlap or expected to overlap by chance if regulation is random.

**Cell line**		**Total DEGs vs parental**	**Overlapping total DEGs**					
			**KI1**		**KI2**		**KI3**	
			**Obs**	**(Exp)**	**Obs**	**(Exp)**	**Obs**	**(Exp)**
RKO	KI1	265	-	-	90	(≤16)		
	KI2	678	90	(≤16)	-	-		
HCT 116	KI1	73	-	-	16	(≤3)	19	(≤4)
	KI2	137	16	(≤3)	-	-	36	(≤5)
	KI3	209	19	(≤4)	36	(≤5)	-	-

DEG – differentially expressed gene; Obs – observed; Exp – expected statistically (upper limit for 99% confidence interval, Bonferroni-correction for multiple hypothesis testing).

Table S3. Upregulated genes in RKO and HCT116 $\mathit{KMT2C}^{insG}$ clones observed to overlap or expected to overlap by chance if regulation is random.

**Cell line**		**DEGs upreg vs parental**	**Overlapping upregulated DEGs**					
			**KI1**		**KI2**		**KI3**	
			**Obs**	**(Exp)**	**Obs**	**(Exp)**	**Obs**	**(Exp)**
RKO	KI1	152	-	-	44	(≤9)		
	KI2	444	44	(≤9)	-	-		
HCT 116	KI1	29	-	-	4	(≤2)	7	(≤2)
	KI2	71	4	(≤2)	-	-	25	(≤3)
	KI3	105	7	(≤2)	25	(≤3)	-	-

DEG – differentially expressed gene; Obs – observed; Exp – expected statistically (upper limit for 99% confidence interval, Bonferroni-correction for multiple hypothesis testing).

Table S8. PCR primer sequences.

Number	Name	Step	Sequence
1	HA1attB1	HA1 amplification for cloning	**GGGGACAACTTTGTATAATAAAGTTG**TGGTGATAGCTTTGCTTTTGT
2	HA1attB4	HA1 amplification for cloning	**GGGGACAACTTTGTATAGAAAAGTTGGGTG***ATTTGCTGAGGAGGCACAAG*TTCGGGGATGATGTTTGAAT
3	HA2attB3	HA2 amplification for cloning	**GGGGACAACTTTGTATAATAAAGTTG**TGGTGATAGCTTTGCTTTTGT
4	HA2attB2	HA2 amplification for cloning	**GGGGACCACTTTGTACAAGAAAGCTGGGTA**CTTCCGTTTTTACCTCATTGG
5	M13F	Entry clone screening	GTAAAACGACGGCCAGT
6	M13R	Entry clone screening	GTCATAGCTGTTTCCTG
7	HA2 mutF	Site-directed mutagenesis	CAGTGTGTATCTGTTGAACCAAAGAAAAAGGAACAAGAAAACAAAAC
8	HA2 mutR	Site-directed mutagenesis	GTTTTGTTTTCTTGTTCCTTTTTCTTTGGTTCAACAGATACACACTG
9	LR HA1F	LR screening HA1	TCCACCACTAGGGGTTCCTG
10	LRHA1R	LR screening HA1	CCTGTGGAGAGAAAGGCAAA
11	LR HA2F	LR screening HA2	GGGTTCCTGCGGCCGCCAATCCG
12	LR HA2R	LR screening HA2	TCGCCTTCTTGACGAGTTCT
13	KI ScrF	Cell clone construct integration screen	CAGGAGGCTGAGACAAAAGG
14	KI ScrR	Cell clone construct integration screen	ATTTGCTGAGGAGGCACAAG
15	HA1 scr F	Cell clone HA1 integration	CAGGAGGCTGAGACAAAAGG
16	HA1 scr R	Cell clone HA1 integration	TCGCCTTCTTGACGAGTTCT
17	HA2 scr F	Cell clone HA2 integration	TCGCCTTCTTGACGAGTTCT
18	HA2 scr R	Cell clone HA2 integration	CAGAAGCCTGTGAGCAAGGA
19	Ad cre F	Selection cassette removal screen	GAGGCCTGTAAGTCCCTCAA
20	Ad cre R	Selection cassette removal screen	CCTCCTGGAAATCCAAATCTT

Bold - Att tag, *Italics* - Screening primer seq tag

Table S9. The shRNA lentiviruses and TaqMan probes used for stable knockdown cell line generation.

Gene	SMARTvector–shRNA lentivirus	TaqMan probe
TSPAN1	TSPAN1-VHS00-6200548<br>TSPAN1-VHS00-7719637<br>TSPAN1-VHS00-9695842	Hs00371661_m1
PRSS23	PRSS23-VHS00-4789171<br>PRSS23-VHS00-8053333<br>PRSS23-VHS00-8515531	Hs00359912_m1
Non-silencing	RHS4348 (GIPZ-Lentivirus)	
Actin		Hs01060665_g1

Table S10. Primers for RT-qPCR with SYBR Green detection.

Gene	Primer sequences	
	Forward	Reverse
ANK1	AAAACGGCTCCGTGTGGAAGGA	GATGATTCGGCACACCCTCTTC
HBE1	AACCTCAAGCCCGCCTTTGCTA	GGTGAACTCCTTGCCAAAGTGAG
HKDC1	ATCGCCGACTTCCTGGACTACA	GCCTTGAAACCTTTGGTCCACC
KCNN4	CATTCCTGACCATCGGCTATGG	GCCTTGTTAAACTCCAGCTTCCG
KMT2C	ATGTGTATCTGGCACGGTCTC	CAATGTCTCGAGCAGCATACA
PLK2	CAACAATGGTGCTCACATGAGCC	GGAGCATCTGTTGCTGGGAAAAC
PRSS23	GTGAGGATGTGGAAGAGACAGC	GAGAGGAGTGATTCTGACAGCC
RPS4Y1	GCCGTTTTGCTGTTCACCGCAT	ATCTGGGTAGCGGATGGTTCGA
SAMD9	GGGAACTACCTTGGCTATGCAC	CGTATTCCTGACGGTTCATTGCC
SUSD2	TGCACTTCGGATCATCGACAGC	TCCTCTCGGAAGTCATCGCTCA
SYK	CGTATGAGCCAGAACTTGCACC	CTTTCGGTCCAGGTAAACCTCC
TESC	CTGAGTGGAGATCAGCCTACCA	CTGTTGTCGAAGAAGGCACGAAC
TM4SF18	GGATACTGCCTGGTCATCTCTG	TGCAGGTTCCAGGCACTGAATC
TNFSF4	CCTACATCTGCCTGCACTTCTC	TGATGACTGAGTTGTTCTGCACC
TSPAN1	TGCTGTGGTCGCCTTGGTGTAC	TGGTGAAGCCACAGCACTTGAG
TSPAN7	CTCATCGGAACTGGCACCACTA	CCTGAAATGCCAGCTACGAGCT
WFIKKN1	GTGTGCCCCAACCAGCTCAGC	CACACGTTGATGCAGCACTTCTC
