# Supplementary material for: Restoration of KMT2C/MLL3 in human colorectal cancer cells reinforces genome-wide H3K4me1 profiles and influences cell growth and gene expression
Source: Clin Epigenetics. 2020 May 29;12:74. doi: 10.1186/s13148-020-00863-z (PMC7257146; doi:10.1186/s13148-020-00863-z)
Supplement: Supplementary file 4 — Additional file 4. Uncropped gels for Figure S1 [file 13148_2020_863_MOESM4_ESM.pdf]

ADDITIONAL FILE 4

Uncropped gels for Figure S1

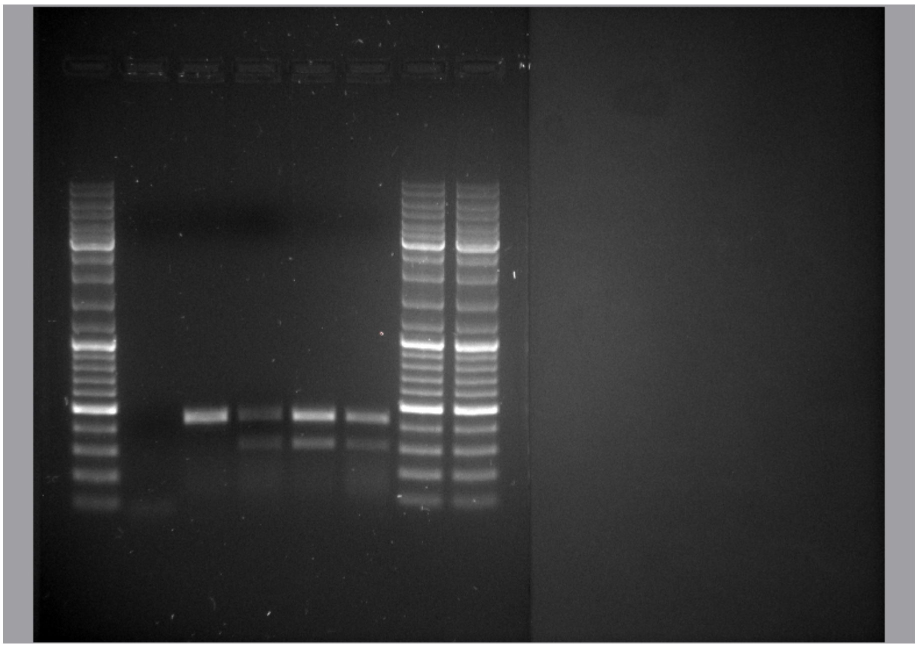

Loading: Ladder, Neg C (H_2O), RKO parental, RKO KI1, RKO KI2, RKO KI2 (independent DNA prep), ladder

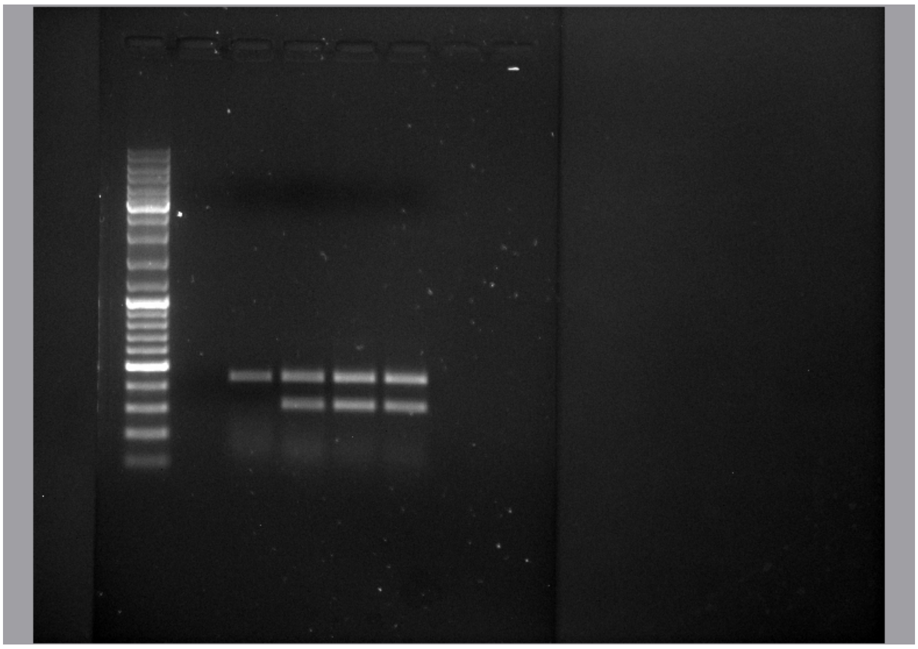

Loading: Ladder, Neg C (H_2O), HCT116 parental, HCT116 KI1, HCT116 KI2, HCT116 KI3
